# The British Nurses Association

**Published:** 1888-03-10

**Authors:** 


### The British Nurses' Association.

We are informed that the bye-laws of this Association, as finally adopted, are as follows :

1. The Association shall be entitled the "British Nurses' Association." 2. Its objects shall be : (*a*) To unite all qualified British nurses in membership of a recognised profession. (*b*) To provide for their registration on terms satisfactory to physicians and surgeons, as evidence of their having received systematic training. (*c*) To associate them for their mutual help and protection, and for the advancement in every way of their professional work. With a view to the attainment of these objects, it shall be the immediate aim of the Association to obtain a Royal Charter incorporating the Association and authorising the formation of a register which shall include all nurses, as described in Bye-law 4, applying for registration before the first day of January next following the granting of the Charter, and such nurses at subsequent dates as shall fulfil the conditions which the Charter may prescribe. 3. The Association shall be composed of nurses and such medical men as may express their desire to become members. 4. Until January 1st, 1889, the following shall be eligible to become members on producing to the Executive Committee satisfactory evidence of professional attainments and personal character, and of having been engaged three years in nursing —viz., the past or present matrons or lady superintendents, sisters, or nurses, of any public hospital or infirmary, or public or district nursing association in the British Empire, all trained midwives, and women who have been engaged in private nursing. 5. On and after January, 1st, 1889, the conditions of membership shall be determined from time to time by the Executive Committee, subject to the approval of the General Council. 6. The members of the Association shall meet annually to elect a General Council, to receive the annual, financial, and other reports for the previous year, and to transact such other business as may be necessary. 7. The General Council shall consist of the President, the Vice-Presidents, 100 medical men, inclusive of the teachers of nursing in metropolitan hospitals, of whose number two shall be *ex-officio* members of the Executive Committee ; 100 past or present matrons, and 100 sisters or nurses, including the members of the Executive Committee. It shall meet once in three months, or oftener if necessary, to receive the report of the Executive Committee for the previous quarter, and to transact such other business as may come before it. The ultimate decision on any matter affecting the Association shall rest with the General Council. 8. At any division in the General Council or Executive Committee, if two members shall so desire, the votes of the nurses and of the medical men shall be taken separately, and no motion or amendment shall be declared carried which does not obtain a majority of votes in each group. 9 The Executive Committee shall consist of the following members :—The President of the Association, Mrs. Bedford Fenwick, the matrons of the general hospitals of the metropolis to which recognised medical schools are attached, two teachers of nursing as provided by Bye-law 7, and the heads of the navy and army nursing departments ; and of the following twenty-seven elected members, viz. : four physicians, four surgeons, four general medical practitioners, four matrons of metropolitan hospitals, general or special, exclusive of those possessing medical schools, two matrons of the poor-law infirmaries of the metropolis, one sister in charge of a ward, one staff nurse at a metropolitan hospital, one lady superintendent of district nurses, four matrons of provincial hospitals or infirmaries in England or Wales, one matron of a Scottish hospital, and one matron of an Irish hospital. 10. The elected members of the Executive Committee shall be appointed by the General Council at its last meeting in every year. The first Executive Committee shall hold office for three years. Thereafter at least one-third of the nursing and one-third of the medical members shall retire annually by rotation, and shall not be eligible for re-election till the following year. 11. The Executive Committee shall have power to appoint all paid officers and to determine what salary each shall receive. It shall initiate and draw up in detail, and refer to the General Council, such measures as may seem advisable from time to time, and shall carry into execution all measures of which the General Council shall approve. And, generally, it shall conduct the business and watch over the interests of the Association. 12. The General Council and the Executive Committee shall respectively frame regulations and bye-laws for their own guidance. 13. The honorary officers shall be a patron, a president, vice-presidents, a treasurer, and trustees, of whom the vice-presidents must possess the qualifications necessary for membership of the Association. As vacancies occur or occasion may arise, they shall be nominated by the Executive Committee and elected by the General Council. 14. The annual subscription for members shall be—for matrons and medical men, half-a-guinea, or a life subscription of five guineas in one sum ; for sisters and nurses, half-a-crown, or a life subscription of one guinea in one sum. All subscriptions shall be due on January 1st of each year. 15. A General Council meeting shall have power to direct the secretary to erase from the roll the name of any member who shall, after full inquiry, be deemed unworthy to remain a member. But no member's name shall be erased for this cause except by the order of a General Council meeting specially summoned to consider the matter, and at which meeting twenty-five shall form the necessary quorum. Provided always that any member whose name has been directed to be erased from the roll shall have the right to demand that, before such erasure takes place, the matter shall be referred to a general meeting of the members of the Association.

We should be glad to hear the views of our readers upon the above scheme. Meanwhile we are requested to state that the representatives of the nurse training schools have had the question of the registration of nurses under consideration. A statement of their views and decision may be expected shortly. Until this is published, no one can form a final judgment on the questions involved, and for this reason matrons and nurses will be well advised if they abstain from giving their money to any association for the present.—Ed. *T. H.*

